# Not a Standalone Treatment: Considerations for Psychedelic‐Assisted Therapy

**DOI:** 10.1002/brb3.71280

**Published:** 2026-04-09

**Authors:** Sharon Murphy, Elizabeth Stewart, Jacqueline Winship, Laura Kampel, Benjamin Stiel, Justine Ellis

**Affiliations:** ^1^ Ramsay Clinic Northside St Leonards New South Wales Australia; ^2^ Black Dog Institute Randwick New South Wales Australia; ^3^ University of New South Wales, Discipline of Psychiatry & Mental Health Randwick New South Wales Australia

**Keywords:** depression, psychedelics, psychotherapy, PTSD

## Abstract

**Purpose:**

Psychedelic‐assisted therapy (PAT) is becoming a clinically available treatment for patients with treatment‐resistant conditions such as depression and post‐traumatic stress disorder (PTSD) in several countries. As PAT transitions from research settings into routine clinical practice, there is a growing need for coherent clinical frameworks and evidence‐based guidelines to support its safe, effective, and integrated delivery. This paper examines how external therapists and psychiatrists can best support patients throughout the PAT journey; the different models for involving external therapists in PAT; the challenges and opportunities associated with collaboration between external therapists and PAT teams; beneficial characteristics of external therapy; and priority areas for future research.

**Method:**

A literature review was conducted, including empirical studies, theoretical papers, and position statements that explored the role of external therapies alongside PAT. This information was integrated with multidisciplinary clinical experiences to develop a series of guiding principles and practical considerations for clinicians.

**Findings:**

There is currently no cohesive framework to guide collaboration or care coordination between external therapists and PAT teams. The review suggests that PAT should not generally be conceptualized as a standalone treatment for patients with treatment‐resistant conditions. External therapists play a critical role across preparation, dosing, and integration phases, contributing to patient safety, continuity of care, and therapeutic integration. Models for involving external therapists in PAT are identified, each with opportunities and challenges. Practical strategies are outlined to mitigate risks and address challenges associated with interprofessional collaboration. Further research is required to refine clinical frameworks and inform best practices.

**Conclusion:**

While scientific and public narratives often portray PAT as a discrete or self‐contained intervention, clinical practice indicates it is most effective when embedded within a broader, integrated therapeutic process. Effective collaboration between external therapists and PAT teams is essential to maximize therapeutic outcomes and ensure patient safety.

## Graphical abstract

External therapists play a pivotal role in supporting patients undergoing psychedelic‐assisted therapy (PAT), offering continuity of care, support, and integration. Co‐operation and collaboration between external therapists and PAT teams is vital to ensure patient safety and optimal treatment outcomes. Further research is needed to establish best practice guidelines.

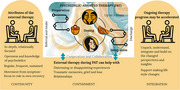

## 1 Introduction

There are a significant number of patients with treatment‐resistant depression and hard‐to‐treat post‐traumatic stress disorder (PTSD) currently under the care of mental health clinicians—including psychiatrists, psychologists, psychotherapists, general practitioners, and other mental health workers (herein referred to as the “existing treatment team”). Significant resources are allocated to this patient population, often with limited or modest results (Al‐Harbi 2012; Bisson and Olff 2021; Heerlein et al. 2021; Kazlauskas 2017; Metcalf et al. 2020). Psychedelic‐assisted therapy (PAT) has recently emerged as a promising intervention for these patients (Kalfas et al. 2023; Ko et al. 2023; Krediet et al. 2020; Reiff et al. 2020; Yao et al. 2024).

PAT has become available for clinical care following the recent down‐scheduling of psilocybin and/or MDMA in Australia, New Zealand, Canada, several countries in Europe and South America, and a number of states in the United States. As the “T” in PAT suggests, therapy is a core part of this treatment process. A recent debate has emerged about whether psychotherapy is necessary alongside the administration of a psychedelic substance (Weintraub and Miklowitz 2024). Although this discussion has garnered attention, it has largely focused on the therapeutic support provided during or immediately surrounding the dosing sessions—typically by the PAT facilitation team (Weintraub and Miklowitz 2024). In contrast, the longer‐term psychotherapeutic work that may precede and follow PAT has received comparatively little attention.

Despite some debate, the safety and efficacy of psychedelic treatment is reported to be improved when a patient has therapeutic support before, during, and after their psychedelic experience (Earleywine et al. 2024; Modlin et al. 2024; Zamaria et al. 2025). A sense of psychological safety and trust with the therapist has been consistently correlated with more positive experiences for patients (Kamilar‐Britt et al. 2023; Modlin et al. 2024; Murphy et al. 2022). Aside from the PAT team, support can be provided by a patient's existing treatment team, with whom they presumably already have a strong therapeutic alliance. An external therapist with knowledge, understanding and openness to their patient's psychedelic experiences increases the likelihood that they will: (a) be adequately prepared for the psychedelic experience, with realistic expectations and coping strategies that enable them to deal with emotional and somatic experiences as they arise; and (b) be able to integrate and make meaning from the experience, process emotions, shift deeply held thought patterns, and form a cohesive narrative to make sense of what unfolds (Gorman et al. 2021). Without adequate support, the patient could fail to capitalize on the therapeutic opportunity or have a negative subjective experience on the psychedelic, or a “bad trip” (Statharakos et al. 2025). Such negative subjective experiences, which may involve anxiety, panic, traumatic flashbacks during the dosing session, and/or intrusive thoughts, have been reported among patients who lack external supports, including having no external therapist engaged (Bhatt et al. 2025; Bremler et al. 2023). Negative experiences have also been documented when patients report reaching out to therapists who have negative beliefs or biases about PAT, or who lack knowledge of psychedelic‐related experiences and symptoms (Bremler et al. 2023). In a narrative review, Statharakos et al. (2025) provide an in‐depth discussion of the nature and complexities of “bad trips,” their psychological implications, and their potential to be either limiting/traumatizing or to promote psychological growth and healing for patients. The authors argue that bad trips, when experienced in a controlled and supportive therapeutic setting, can be valuable tools for healing, but that without such support may remain stuck as overwhelming feelings of fear, confusion or distress (Statharakos et al. 2025).

While quite a few clinician‐focused professional development courses are already in circulation, which are designed to educate clinicians about PAT, there may be a lack of awareness of these programs or motivational, time, or financial barriers to accessing them. In addition, there may be a misconception that, if one is not planning on being a PAT therapist, then there is little benefit in learning about PAT. However, sufficient knowledge and understanding of psychedelic experiences and PAT protocols among psychotherapists who are not directly working with altered states, but who are supporting patients undergoing adjunctive PAT, is vital (Negrine et al. 2025). Greater uptake of these programs is also likely to improve communication and collaboration between the existing treatment team and PAT team, which is critical to the progress patients make (Hultgren et al. 2025). To date, there is little evidence about how the existence, nature, length or frequency of external therapy, and the quality of collaboration between the external therapist and the PAT team, impact treatment outcomes (Hultgren et al. 2025).

This paper proposes that to achieve sustained improvements in a treatment‐resistant patient's mental state, thinking patterns, emotional stability, and functioning in everyday life, PAT cannot be viewed as a standalone treatment, but as an adjunct to their longer‐term psychotherapeutic work. In order for PAT to work effectively as an adjunct treatment, the existing treatment team requires information about: (a) PAT protocols; (b) the varied needs of patients before, during, and after PAT; (c) potential models of care for inclusion of/collaboration with the external therapist; and (d) the challenges that can arise when working adjunctively in this space and how to manage them. This paper is a helpful starting point to address these areas and, in addition, provides initial considerations about the nature, frequency, duration, and type of external therapy that could enhance the overall effectiveness of PAT.

## 2 PAT Protocols

**FIGURE 1:**
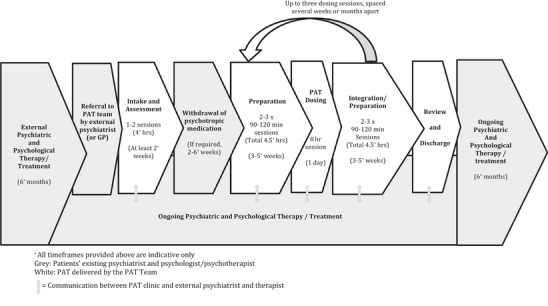
Proposed model of care for psychedelic‐assisted therapy (PAT) with external treatment team.

Protocols for PAT generally involve three components: preparation, medicine administration, and integration. This bundled treatment is what has been termed psychedelic‐assisted therapy (Chisamore et al. 2024; Palitsky et al. 2023; Weintraub and Miklowitz 2024). While protocols vary between clinics, they typically involve between one and three medicine dosing days, preceded and followed by two to three preparation and integration sessions, respectively (Figure 1). The total number of patient contact hours with the PAT team may be between 16 and 42 h or more, across 2 to 12 months or longer. Figure 1 illustrates a typical PAT treatment protocol, as well as how PAT might ideally sit within an existing treatment framework. In Figure 1, the existing treatment team is represented in gray, the PAT team in white, and communication between teams by the five small arrows spanning the white and gray steps. Much attention has already been given to the processes involved during PAT itself (the steps shown in white) (Chisamore et al. 2024; Weintraub and Miklowitz 2024). This paper will therefore focus on the steps shown in gray in Figure 1, that is, the treatment carried out by the existing treatment team. Of relevance to how external therapists are involved, which will be discussed in greater detail further in this paper, is that PAT protocols usually specify that two PAT‐trained mental health clinicians, collectively referred to as the “PAT dyad” or “PAT team,” attend the preparation and integration sessions, and dosing day.

## 3 External Therapeutic Support for PAT

Given the extensive contact hours between the patient and the PAT team, it may be assumed by some clinicians that the patient would have a break from their external therapy while undergoing PAT. However, because of the intensity of affective states, thoughts, and the nature of memories that may arise during psychedelic experiences, having the support of an external therapist is important (Weintraub and Miklowitz 2024). Where no external therapist is engaged prior to the PAT referral, the PAT team may, during assessment, consider recommending a suitable external therapist.

### 3.1 Before

Preparation is a crucial phase of psychedelic‐assisted therapy (PAT), fundamental to the safety and efficacy of the process (Johnson et al. 2008). The PAT team is focused on guiding the patient toward a comprehensive readiness to undertake PAT (Phelps 2017). The external therapist can also play an important role during this phase. Effective preparation is about helping patients to:

- Manage physical sensations and feelings that arise when withdrawing from their psychotropic medications, if applicable (Johnson et al. 2008; Phelps 2017).
- Cultivate readiness for unfamiliar and potentially frightening experiences that may unfold during the dosing session (Phelps 2017; Watts and Luoma 2020).
- Teach grounding and regulation skills to assist with challenging physical sensations, emotions, memories, imagery, or perceptions that may emerge during dosing  (Phelps 2017).
- Explore with patients how they might relate to whatever arises with openness and acceptance, rather than resistance (Carhart‐Harris et al. 2018).
- Identify ambivalent parts of self to ensure genuine consent for the process (Watts et al. 2017).

Adopting a “hopeful but realistic” stance is key, expressing hope while maintaining a balanced perspective of the range of possible outcomes, and avoiding unrealistic expectations of a “miracle cure.” While there can be significant pharmacological benefits of the medicines, it is unhelpful if a patient sees themselves as a passive participant.

The PAT team's role includes an exploration of intentions and motivations (McAlpine et al. 2024), and the external therapist can also assist with this in parallel, demonstrating that they are engaged and prepared to come alongside the patient in this journey. “Intention setting” is a central task in preparing a patient for PAT, and serves a distinct clinical purpose, often requiring a shift from identifying with symptoms or blaming circumstances toward acknowledging personal responsibility for change (Johnson et al. 2008; Carhart‐Harris et al. 2018). In discussing the therapeutic intention, the external therapist and patient can also explore both the “why?” and “why now?” for undertaking PAT, rather than focusing on expectations about what will happen. Clearly defined intentions act as anchors during a psychedelic experience, supporting constructive engagement with challenging material and helping to develop a shared narrative of the therapeutic journey (Kettner et al. 2019). As insights deepen, intentions may evolve, continuing to orient the patient's process, and the external therapist can be a part of continually updating and revising intentions. Research suggests that clear intentions foster more meaningful experiences and strengthen commitment to healing (Haijen et al. 2018). In this way, intention setting becomes a powerful therapeutic tool—helping patients show up fully, co‐author their recovery, and cultivate positive expectancy for change (Phelps 2017).

### 3.2 During

While the external therapist is not usually involved in the medicine administration day or integration sessions (unless they are part of the PAT dyad), it can be helpful for a patient to see their external therapist between the dosing and integration sessions, as the process of PAT can be destabilizing for patients. This additional support also allows the external therapist to hear about the patient's journey in immediate proximity, enabling them to draw on the color, emotion, and intensity of the experiences in the external therapy. Things that might come up during the process include (but are not limited to):

- Challenging physical and psychological experiences and/or feeling distress after the dosing day and/or a “bad trip.”
- The perception that “nothing happened”; feeling angry and robbed of a “real experience.”
- Historic trauma is emerging and being processed in a different way.
- The lost time due to the illness may come into sharp focus, and the patient may experience a grief reaction.
- Re‐appraisal of close relationships in both positive and negative ways.
- New insights emerging that may need extended therapy to integrate.
- Spiritual or existential experiences or intergenerational insights.

In PAT, challenging physical and psychological experiences may be therapeutically useful, but may also be viewed negatively by the patient. Sometimes, the difficulties arising in PAT are reflections of the core issues that challenge patients in their lives. Working through such experiences can provide valuable sources of insight and growth and can be used to aid understanding and change.

While psychedelic teams do provide patients with therapeutic support between dosing days, external therapists can provide additional support, such as holding hope when the patient feels discouraged, helping to frame difficult experiences as part of a nonlinear healing process, or reminding the patient of the importance of integration work that can take months. This scaffolding often plays a vital role in helping a patient navigate the inevitable highs and lows of the process.

### 3.3 Aftercare

Although patients receive several hours of integration therapy at the PAT clinic following the dosing day, it is not realistic to expect that these patients, who have often been struggling with mental illness for years, will achieve a full recovery during the PAT timeframe. PAT is an adjunct to the patient's longer‐term therapy, and it may take many months and even years for a patient to fully unpack, understand, integrate and build on the changed perspectives and insights from PAT into their life. For many of these patients, they may have lost employment, friends, key relationships, and/or engagement in life, and they will need psychological support to process and rebuild their lives.

Aftercare may involve assisting patients to continue integrating the PAT experience and its impact on their sense of themselves and their lives. They may benefit from reflecting on their initial intentions and the way that these have evolved or changed throughout the PAT experience. Furthermore, given the frequent presence of spiritual, existential, religious, and theological (SERT) experiences in PAT (Hartogsohn 2018; Palitsky et al. 2023), processing these may form an important component of aftercare. Therapists need to be aware of their own cultural and/or religious biases and take care to respond to SERT content in a culturally sensitive manner that respects the patient's own value system. SERT content may have been experienced by the patient as confronting, comforting or inspirational, depending on their religious and cultural framework. Working through the experience from within the patient's own frame of reference may present possibilities for positive meaning‐making and transformation.

## 4 The External Therapist's Involvement in PAT

There has been a debate about how to best include external therapists in PAT treatment. In a recent paper on the quality of the patient‐therapist relationships in PAT, Villiger (2025) outlined two possible scenarios for involvement of external therapists. In the first, the external therapist participates as one of the two therapists in the PAT dyad, and is present for preparation, dosing, and integration. In the second scenario, the external therapist is not present during the psychedelic experience but can be included in debriefing and is provided with summaries of the experience. At present, the second scenario—where the external therapist is not included in the psychedelic sessions—may be the more commonly adopted model in many countries due to high costs of treatment, availability of PAT therapists, and overall feasibility of the model. Despite this, it may not be the best model for all patients. Circumstances where involvement of the external therapist in psychedelic dosing is more likely to be considered include: (1) when the external therapist is already a current or recent member of the PAT team, and/or (2) if the patient and external therapist are both First Nations people, or from another Culturally and Linguistically Diverse group. In these cases, the richness that an external therapist brings in helping the patient feel safe may outweigh the complications of setting up a new working relationship. Overall, there are opportunities and challenges of each approach to involving the external therapist, which are outlined in Table 1.

**TABLE 1:** Opportunities and challenges of including an external therapist in the PAT dyad.

Opportunities for including the external therapist in the PAT dyad	Challenges of including the external therapist in the PAT dyad
● Existing therapist has established a strong therapeutic alliance and trust with the patient. ● Existing therapist holds significant autobiographical information about the patient as well as a conceptualization of their core struggles, anxieties and defenses. ● Existing therapist presence increases their ability to weave aspects of the psychedelic experience into the patient's external therapy. ● For patients who identify as Indigenous or from a Culturally and Linguistically Diverse Group, the presence of their external therapist may increase safety and readiness for the psychedelic experience, as well as facilitate culturally appropriate interpretation and meaning making with the patient.	● Moving from a traditional therapy setting (i.e., therapist and patient in chairs sitting across the room from one another) to a PAT setting (patient moving from a bed to sitting to walking) can be disruptive to the established therapeutic process and changes the frame of the therapy. ● Time commitment and scheduling of PAT may make it difficult for external therapist to be available (time commitment also involves liaising with PAT therapist). ● PAT work may involve reduced remuneration for the external therapist. ● PAT dyads need a good understanding of the partner's model of therapy and therapeutic style. PAT teams spend considerable time cultivating the dyad partnership (e.g., supervision, multi‐disciplinary team meetings, attending trainings together). ● A lack of this preparatory work for the PAT dyad could negatively impact the therapy (e.g., conflict or tension in the room). ● Medicolegal implications for involving an external therapist in PAT are unclear. ● Lengthy and costly background checks, onboarding, inductions, and OH&S trainings may be required.

Penn and Yehuda (2023) question to what extent clinical practice will need to conform to the protocols used in clinical trials. For example, there is a third option where the patient's therapist provides PAT at some point in a long‐term therapy process. Such a model has not been tested in research trials, and, in most countries, is not an approved protocol at this stage. While such a model of treatment might be approved in the future for certain populations, initial referrals in Australia have generally been for patients with severe, complex mental health conditions and often with physical health conditions that require monitoring during dosing, and so this population is currently best suited to dosing with the safeguards in place at PAT clinics. Receiving PAT in an unfamiliar clinic setting can be challenging for certain vulnerable subgroups, including individuals with borderline personality disorder and complex post‐traumatic stress disorder (cPTSD). This can generally be managed by offering some patients an increased number of preparation and integration sessions to support the development of trust with the PAT team and to carefully manage endings. The support from the external therapist is important to provide containment and continuity of care, and collaboration between the PAT team and the external therapist is even more important for this population.

## 5 Challenges of Working Adjunctively

There is little research on the advantages and challenges of working adjunctively. It is common for a patient to be seen concurrently by both a prescribing psychiatrist and psychotherapist, in which issues of communication, accessibility, boundaries and respect arise between the professionals and yet there is minimal research on this “split treatment model” (Gitlin and Miklowitz 2016).

Similarly, most therapists will have had experiences of patients undertaking some form of adjunctive therapy, such as concurrently seeing a family therapist or couple's therapist. While challenges do still arise, generally, the therapist's contact with these other practitioners is minimal and the focus of work does not significantly overlap.

In contrast, PAT typically involves many hours of therapy over an extended period of time and the therapeutic focus can overlap with the therapist's focus, creating a new landscape for therapists to navigate. Practical challenges such as scheduling therapy sessions around the psychedelic protocol may arise, with existing therapists being asked to move their appointment to an alternative day to facilitate PAT appointments. In addition, complex professional jealousies may arise and a sense of one's therapeutic relationship being impinged upon. The existing therapeutic relationship may be long‐standing, and it can be difficult to “share” one's patient with another therapeutic team. The PAT team is also working with a patient while they are under an empathogen, or an otherwise powerful medicine, potentially quickly strengthening their therapeutic alliance. Under these conditions, the therapist can feel sidelined or undermined. These challenges may be heightened if a patient returns from PAT with new insights, different ways of understanding themselves, new language to describe their internal experience, or a sense of having made rapid progress during the process. One way to mitigate these issues is for PAT teams to be sensitive to these challenges for the external therapist. Another helpful framing is that when meaningful progress occurs during PAT, it should not be viewed as distinct from the preceding therapeutic work, but rather as evidence that a patient has been able to build on the foundation of their existing therapy to fully engage with PAT, which in turn can result in an acceleration of the external therapeutic process.

In comparison with other adjunctive therapies, PAT tends to require a greater exchange of information between an external therapist and the psychedelic team. The therapeutic space is traditionally very private and insulated. The communication required between an external therapist and the PAT team may feel unfamiliar, uncomfortable, and time‐consuming. However, PAT teams may value and benefit from insights and conceptualizations shared by the external therapist, and vice versa, as these help to shape and support the patient's experience. In addition, the patient may feel unable to provide a concise summary of the 8‐h dosing day to their therapist in a 50‐min therapy session. Summaries from the dosing day, including images, insights and anxieties arising in the psychedelic treatments provided by the PAT team to the external therapist, can be valuable.

Another challenge of adjunctive work is the risk of “splitting,” whereby the patient devalues the therapist and idealizes the psychedelic team, or vice versa. Such changeable dynamics can undermine the full therapeutic benefit of PAT, while also highlighting the patient's patterns of relating. The emergence of these issues can create challenges, as well as opportunities for growth, when explored with a patient. Supervision and timely communication between an external therapist and the psychedelic team can help to pre‐empt and manage these relational challenges, thereby interrupting splitting.

A range of communication methods have been used to date in the Australian context, determined on a case‐by‐case basis, including phone calls and case conferences between the external therapist and the PAT team. In other situations, a therapeutic letter has been written by the PAT team with input from the patient for the external therapist. To facilitate timely communication, PAT teams could develop a template for information sharing with the external therapist, with a PAT mental health nurse being assigned the role of managing this communication. By way of example, see Table 2 for a communication template that could be used for two‐way communication between the PAT team and the external therapist.

**TABLE 2:** Example of two templates to facilitate communication between psychedelic‐assisted therapy therapist and existing therapist.

At time of referral: Communication from existing therapist/psychiatrist to the psychedelic‐assisted therapy therapists re [patient name]
1. A summary narrative ^1^: Basic biographical information about the patient from early life and family of origin through to current situation. Basic treatment history and diagnosis, and predisposing, precipitating, and perpetuating factors. Resources/strengths of the patient and the family/social and therapeutic systems around them. Perpetuating factors to consider: · ongoing stressors in patient's life; · psychological conflicts, avoidance, secondary gains, problematic coping mechanisms, developmental difficulties/deficits/inadequate resources (e.g., underdeveloped emotional regulation skills and/or low distress tolerance); · personality organization (i.e., traits), such as rigidity, sensitivity, and masochism, and possible or diagnosed co‐morbidities (e.g., neurodivergence, OCD); · family dynamics and social network; · ungrieved/incompletely grieved losses; · unprocessed/incompletely processed trauma; · limited access to sufficiently sustained therapy needed to safely process trauma and associated shame, guilt, sadness, and anger, or challenges forming a therapeutic alliance—whether due to the absence of an internalized template of a safe and reliable relationship or due to other reasons. Recent events and recent treatment leading to the referral for PAT.
2. In five years, the patient would like the following to be different in their lives ^2^: [Change timeframe to suit patient.]
3. The attributes/qualities/skills/behaviors/beliefs/thoughts/feelings that the patient would need to acquire/develop/change within themselves to achieve this ^2^:
4. Why is patient drawn to PAT, and why now:
5. The patient's treatment goals for PAT^2^:
6. Does the existing therapist (and treatment team) support PAT for this patient at this time:
7. Self‐regulation strategies, supports, resources, and current safety plan: (including information about their relationship to their support person)
8. Existing therapist's contact details/preferred contacting method:
9. Date form completed:
*^1^ The emphasis on perpetuating factors represents an acknowledgement that PAT is currently reserved for treatment‐resistant conditions in some countries, and therefore, there's a need to consider why the condition is resistant to treatment*. *^2^ These questions promote a shift from a focus on symptoms or external or historical circumstances toward cultivating personal agency for change*.

After dosing and integration 1, 2, or 3: Communication from psychedelic‐assisted therapy therapist(s) to existing therapist/team re [patient name]
1. Intention for dosing day journey:
2. Emotional regulation tools discussed/practiced, modalities/terminology used by this PAT dyad with the patient:
3. Dosage given:
4. Experience and themes on dosing day journey:
5. The significance and meaning of the experience to the patient:
6. Integration techniques used to date:
7. Key takeaways from the experience to date (including any reframing of struggles and path to recovery):
8. Things that have arisen, to date, that might still require additional work:
9. External factors that might be currently challenging or helpful (re parents/partners/etc.):
10. Medications that were withdrawn for treatment. Medications that were continued (if any). Any new medication prescribed or recommenced, or future medication suggestions/considerations:
11. Outcome impression/measures; side effects; safety plan (including any drop in mood or increase in risk):
12. How the patient plans to continue to integrate the experience, any changes patient plans to make in their life, activities the patient plans to introduce or increase:
13. How the external therapist can assist with integration:
14. Schedule of past and future (if any) PAT appointments:
15. Psychedelic‐assisted therapy team and contact details:
16. Date form completed:

Over time, artificial intelligence note‐takers may assist in summarizing preparation and integration sessions, as well as the dosing day into communications, as shown in Table 2.

## 6 Recommendations for External Therapy alongside PAT

Based on the small amount of existing evidence and clinical experience, the following guidance is offered as a starting point for the type, length, and duration of external therapy that may be supportive alongside PAT. These recommendations are offered with an appreciation of the diversity of approaches, views, and ways of working of external therapists.

### 6.1 Type of External Therapy

While the current evidence base is too limited to provide definitive or conclusive guidance on the type of external therapy that is most supportive or beneficial alongside PAT, some tentative suggestions can be made based on emerging narrative‐type research articles and anecdotal evidence from researchers and clinicians working in the field. The forthcoming suggestions are presented with an acknowledgment that rigorous data supporting the efficacy of any specific therapeutic modality to accompany PAT is lacking, and is an area requiring more empirical research. Moreover, we acknowledge that there are considerable financial barriers to accessing both PAT and the external therapy to support PAT for many patients, especially in some countries where out‐of‐pocket expenses are not currently subsidized by either public/government bodies or private insurers. Cost and affordability considerations are further briefly discussed later in the paper.

Weekly to fortnightly therapy, which is relationally focused, and with a therapist holding a favorable view and some understanding of PAT, may be most beneficial. Relationally‐focused therapists typically utilize the therapeutic relationship as an agent of change, and they are skilled in exploring the dynamics between the patient's inner world and interpersonal patterns. These therapists often integrate concepts from a range of modalities, including, psychoanalytic psychotherapy, which use metaphor, projection, exploration of the unconscious, and understanding of defenses and resistance to facilitate insight and emotional processing; parts‐based therapies (approaches like internal family systems (IFS) or schema therapy) that help individuals recognize, dialogue with, and integrate different aspects or “parts” of the self; mentalization‐based therapy (MBT) that enhances the capacity to understand one's own and others’ mental states, improving emotional regulation and interpersonal functioning; art and creative therapies, utilizing creative expression to access and process emotions and experiences that may be difficult to articulate verbally; somatic therapies, focusing on bodily sensations, movement, and awareness to process trauma, release tension, and integrate psychological and physiological experiences; and mindfulness and contemplative approaches, encouraging present‐moment awareness and non‐judgmental observation of thoughts, emotions, and bodily states. Being comfortable working with intense affective states, trauma reprocessing, and the nature of memory may also be beneficial (Phelps 2017).  A level of curiosity and comfort discussing spiritual, existential, and intergenerational material may also be crucial at times. Therapy of this nature tends to lead to a strong therapeutic alliance with an external therapist, which creates a template for establishing trust, an ability to share feelings and receive feedback, compassion, and support—a template the patient may utilize to facilitate a therapeutic alliance with the PAT dyad.

### 6.2 Length and Duration

Ideally, therapy could commence at least 6 months prior to PAT, continue during PAT, and extend for at least 6 months after PAT, or longer. That said, there will be exceptions where patients require more or less extensive preparation and integration than suggested here. Moreover, the costs of such treatment must be taken into consideration, with realistic and feasible treatment plans made with each patient based on their individual circumstances.

### 6.3 External Therapist Training

At present, there is no specific, additional training or approach that is required for an external therapist supporting a patient undergoing PAT. In fact, the nature of work with patients undergoing PAT is not vastly different from how many non‐PAT clinicians already work. There has been some evidence that the ability to support patients undertaking psychedelic treatments is improved when the therapist has had a personal experience with psychedelics (Nielson and Guss 2018). However, there are only a few countries where this is legally accessible, and it is certainly not a requirement to undertake this work, but curiosity and understanding of PAT may be crucial.

Literature reviews suggest that the quality of the therapeutic relationship is particularly important in psychedelic work (Phelps 2017, Murphy et al. 2022). Key competencies that have been proposed for PAT therapists may be useful for external therapists as well (Phelps 2017, 2019). For example, having basic respect for a patient's autonomy, using active listening skills, and focusing on the patient's current concerns is undeniably beneficial (Kamilar‐Britt et al. 2023). Expanding on this, Phelps (2017) outlined six core competencies for PAT therapists, which may be of use for external therapists. These are: empathetic presence; building trust; existential and spiritual intelligence and familiarity with non‐ordinary/mystical states of consciousness; knowledge of the physical and psychological effects of psychedelics; therapist self‐awareness and ethical integrity, for example, boundary setting, knowledge of attachment theory, transference/countertransference processes, self‐care; and proficiency in techniques such as somatic, felt‐sensing/focusing, meditation, music and art therapies.

## 7 Economic Considerations: Costs and Affordability

Cost and affordability of psychedelic‐assisted therapies are a critical consideration. The ability to fund therapy not only affects access to the psychedelic treatment itself, but also the feasibility of engaging in external therapy that is so important alongside PAT. Costs of PAT when delivered in an individual setting have been estimated to range from USD$3000 to $20,000 in the United States (Avancena et al. 2025), 6100 to 7700GBP in the United Kingdom (McCrone et al. 2023), and upward of AUD$30,000 in Australia (Mihalopoulos et al. 2023). Economic modeling suggests that these costs would be substantially reduced, by up to 50%, if PAT were delivered in a group setting (Marseille et al. 2023). Nevertheless, many patients in need of PAT are likely to find current costs financially out of reach, compounded by the fact that external therapy is not bundled into these treatment costs and therefore is an additional fee to patients.

Financial barriers must be considered within the broader regulatory and health‐economic contexts in which PAT is being delivered, which vary between countries. Analyses on the economic costs and benefits of PAT are beginning to show financial benefits of PAT compared to standard care, both for individuals and health systems at large (Avancena et al. 2025). In some countries, such as Australia and countries in the European Union, private and government‐based or public funding is able to be used to subsidize PAT for certain categories of patients (Madero et al. 2026; Mihalopoulos et al. 2023).  Nevertheless, for most people in most countries, PAT is currently unaffordable. Given the emerging nature of the evidence base, more comprehensive analyses of cost–benefit, cost‐effectiveness, and budget impact associated with the implementation and scale‐up of psychedelic‐assisted therapies are still required (Avanceña et al. 2022; Marseille et al. 2022). Such analyses, alongside future research, will be critical in determining whether sustainable funding models can support not only the delivery of PAT itself, but also the provision of external psychotherapy, which is often essential to maximize therapeutic benefit and manage clinical risk.

## 8 Recommendations for Future Research

As mentioned in the introduction, there is a debate about how much or whether therapy contributes to change following use of psychedelic medicines (Weintraub and Miklowitz 2024). In a recent systematic review, Hultgren et al. (2025) found this to be a limitation in the existing PAT literature and suggested the need for more rigorous research.

**FIGURE 2:**
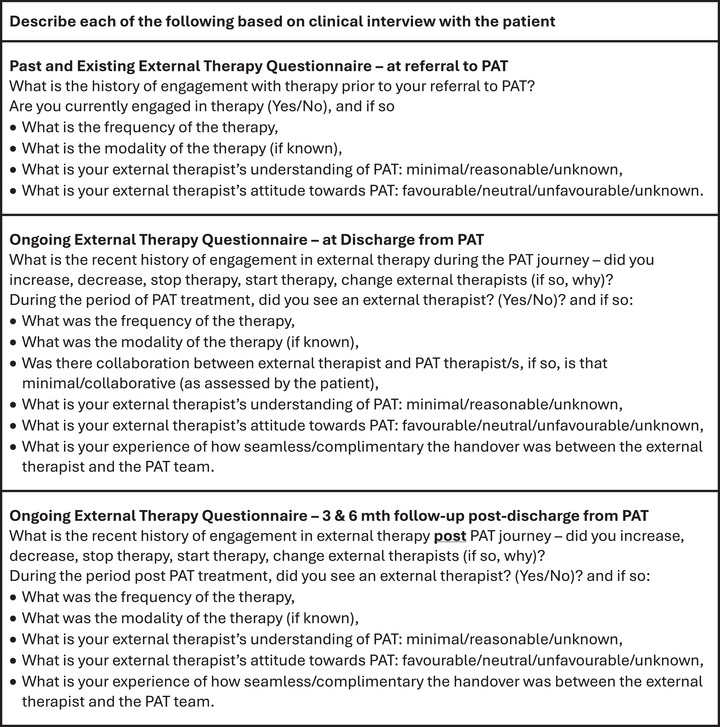
Example questions for inclusion in design of external therapy questionnaires (ETQ).

There is even less research on whether external therapy alongside PAT contributes to improved outcomes, although clinical experience suggests that it does. Likewise, there is no current consensus regarding the external therapy modalities that may be most beneficial or the optimal treatment durations. Many current clinical trials provide minimal or no information about the role of external therapy provided alongside PAT, resulting in a lack of quantitative and qualitative data on external therapeutic modalities, lengths of treatment, and treatment settings. Future research could bridge this gap by routinely collecting data on a patient's external therapy. There may be scope for the design and psychometric evaluation of an external therapy questionnaire to gather standardized data at referral, discharge, and follow‐up. Figure 2 provides example questions that could be used as part of an external therapy questionnaire.

In addition, surveys of attitudes toward psychedelics among mental healthcare providers (Nadeem et al., 2025) could now be extended to ask questions such as, “Have you had a patient who has undergone adjunctive PAT?” and “If so, in your opinion, what was the impact on the progress of the therapy?”

Finally, more research is needed into the economic costs and benefits of PAT in countries around the world, including costs of external therapy, as required. Areas of priority include cost‐effectiveness and cost‐benefit analyses that determine value for money to payers and society at large, scale‐up models that portray the cumulative impact of access to psychedelic therapies, and market analyses that help to plan the delivery of care (Marseille et al. 2022).

## 9 Conclusion

Optimizing the subjective experience and clinical outcomes of patients undergoing PAT is of critical importance, given the significant emotional, psychological, spiritual, and financial investment involved. Negative outcomes may not only be a clinical safety issue, but also a missed opportunity for meaningful therapeutic progress. Psychedelic compounds enhance neuroplasticity and psychological flexibility, providing a unique window to “unlock” emotions and shift psychological defenses and rigid thought patterns that may otherwise remain entrenched or difficult to achieve in a standard talk therapy session. Many patients currently considered for PAT are experiencing severe mental health difficulties and have often not responded to multiple prior treatments. Collaborating and communicating with a patient's external therapist—someone with whom the patient already has a strong therapeutic alliance and a sense of psychological safety and holding—may optimize the overall PAT experience.

## Author Contributions

S. Murphy: conceptualization, writing – original draft preparation, writing – review and editing. E. Stewart: investigation, visualization, writing – original draft preparation, writing – review and editing. J. Winship: conceptualization, writing – original draft preparation, writing – review and editing. L. Kampel: conceptualization, writing – review, and editing. B. Stiel: conceptualization, writing – original draft preparation for section with heading “Before.” J. Ellis: supervision, conceptualization, writing – review, and editing.

## Funding

The authors have nothing to report.

## Conflicts of Interest

The authors declare no conflicts of interest.

## Data Availability

Data sharing is not applicable to this article, as no datasets were generated or analyzed during the current study.
